# Mandibular Body Reconstruction Utilizing a Three-Dimensional Custom-Made Porous Titanium Plate: A Four-Year Follow-Up Clinical Report

**DOI:** 10.1155/2022/5702066

**Published:** 2022-02-25

**Authors:** Carlos-Martín Ardila, Yuritza Hernández-Arenas, Efraín Álvarez-Martínez

**Affiliations:** ^1^Titular Professor University of Antioquia, Medellín, Colombia; ^2^University of Antioquia, Medellín, Colombia

## Abstract

A clinical case of a 42-year-old woman patient, who had a mandibular reconstruction utilizing a three-dimensional (3D) custom-made porous titanium plate dental restoration, is presented. She showed a recurrence of a unicystic ameloblastoma involving the left hemimandible. The patient declined to be managed by a bone-free flap. A mandibular resection in the healthy areas was provided, followed by reconstruction utilizing a 3D custom-made porous titanium plate dental restoration with a hybrid dental prosthesis. The 3D rehabilitation was created considering slim tomodensitometric sections. The cutting guides and custom-created 3D plate were fabricated employing medical software via computer-aided design and fabricating with locations planned for healing abutments. The patient was contented with the rehabilitation, and the condition continued stable at the four-year follow-up.

## 1. Introduction

Bone free flaps are the standard treatment approach for the reconstruction of significant mandibular defects; thus, the main bone sources include the scapula and iliac crest [1]. The recommended technique for mandibular reconstruction is the pedicled flap plus a titanium megaplate [2]. Although this method offers the filling tissue required and specific support, distinct limitations have been described: the establishment of a new surgical place, megaplate exposure or fractures, complications with the articulation, and several esthetic consequences [3–5]. Recently, three-dimensional (3D) custom-made porous titanium plates have been used for mandibular reconstructions, which offer an alternative in those cases of free flap contraindications or refusal by the patient; however, only very short-term follow-ups have been reported. This case report presents a mandibular reconstruction including a custom-made porous titanium plate with a hybrid dental prosthesis followed for 4 years.

## 2. Case Report

**Figure 1:**
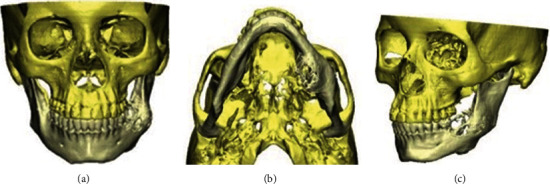
3D reconstruction showing ameloblastoma. (a) Frontal view. (b) Sagittal view. (c) Axial view.

**Figure 2:**
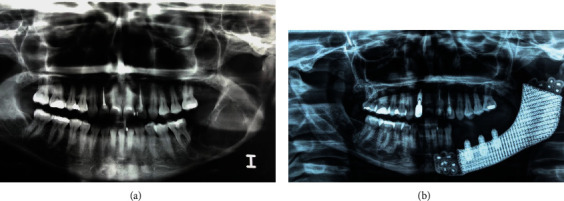
Preoperative (a) and postoperative (b) radiographs.

A 42-year-old woman showed a recurrence of a unicystic ameloblastoma involving the left hemimandible (Figures 1(a)–1(c)). She was intervened twice, with an interval of six years. The two operations included a curettage of the neoplasm cavity down to the apparently healthy bone tissue. She declined to be managed by a free flap. Thus, a mandibular resection in the healthy areas was chosen and provided, followed by reconstruction utilizing a 3D custom-made porous titanium plate with a hybrid dental prosthesis. Preoperative and postoperative radiographs are shown in Figure 2. Written consent of the patient according to ethical principles was signed.

**Figure 3:**
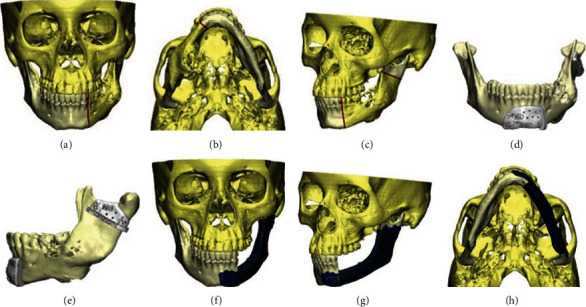
(a–e) Three-dimensional simulation cutting guides. (f–h) Simulation of the custom implant in porous titanium.

The 3D rehabilitation was created considering slim tomodensitometric sections (Figures 3(a)–3(e)). The cutting guides and custom-created 3D plate were fabricated employing medical software via computer-aided design and fabricating (CAD-CAM) with locations planned for healing abutments, followed by the implant sustained prosthesis (Figures 3(f)–3(h)). The titanium plate was designed to allow the placement of dental implants (4.1 mm × 10 mm) at the same time. The designed titanium plate (thickness of 12 mm) was printed with medial grade powder (constituted of porous grade 2 titanium).

**Figure 4:**
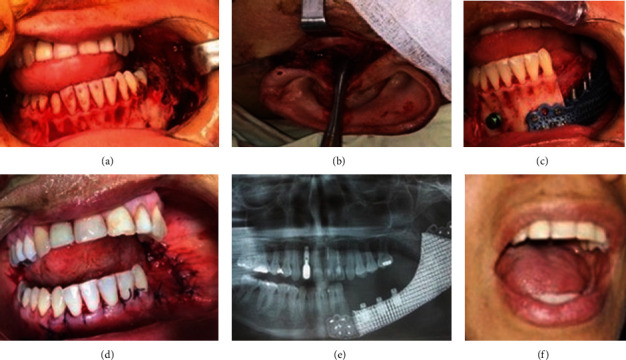
Surgical procedure. (a) Intraoral approach. (b) Preauricular approach. (c) Plate in position. (d) immediate postoperative. (e) Radiograph view at four years postoperative. (f) Intraoral view at four years postoperative.

The surgery was completed under general anesthesia and nasotracheal intubation. The surgical method was intraoral and preauricular (Figures 4(a) and 4(b)). The cutting guides were fixed considering the formerly described markers at the level of the left mandibular condyle and the symphyseal area. The plate was subsequently retained in position using screws for fixation (Figure 4(c)).

Three healing abutments were connected to the porous titanium plate substituting the mandible (Figure 4(c)). The hybrid dental prosthesis was made and attached to the porous titanium plate after two months following the operation. Moreover, miniconical abutments were used to support hybrid prostheses. A metal framework was created for the mandibular hybrid prosthesis, and the definitive prosthesis was completed by means of standard procedures.

**Figure 5:**
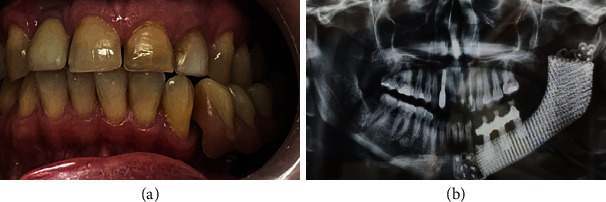
Intraoral view and radiograph after prosthesis.

The patient was given postoperative cleaning guidelines and educated about regular maintenance and possible difficulties associated to the prosthesis. The patient was recalled after 2 days to examine satisfaction and was followed-up further at regular 3-month intervals a year. At all recall intervals, the prostheses were removed, and soft tissue condition was perceived. The patient was satisfied with the rehabilitation, and the condition continued stable at the four-year of follow-up (Figures 4(d) and 4(f)). Intraoral view and radiograph after prosthesis are shown in Figure 5.

## 3. Discussion

The current case presents a mandibular body reconstruction utilizing 3D custom-made porous titanium plate with a hybrid dental prosthesis. The 3D scheme permits respect for the anatomy, and the intervention is considerably shortened than other approaches with the application of the cutting guides. Additionally, this design warrants an impeccable rearrangement of the inferior border and, subsequently, the competence to rebuild a quality mandibular form in contrast with traditional mega-plates [3]. Moreover, the porous titanium permits its tissue ingrowth from the adjacent tissues allowing improved integration [6]. Interestingly, its mechanical strength was equated to cortical bone [7, 8].

To our knowledge, only a few publications have presented 3D custom-made porous titanium plates for mandibular reconstructions; additionally, follow-ups of more than 2 year have not been reported. Qassemyar et al. [3] described two cases demanding reconstruction of the mandibular body, but for whom a bone-free flap was contraindicated. Those patients had a history of ameloblastoma and squamous cell carcinoma. Touré and Gouet [5] treated a patient with a massive recurrence of ameloblastoma of the right hemimandible. The period of patient's follow-up was up to 18 months and was treated with an implant-supported prosthesis. Rachmiel et al. [9] documented a case of a patient with a large deficiency in the ramus. One-year follow-up revealed an appropriate function and form. Lee et al. [10] showed a case with a history of squamous cell carcinoma on the left floor of the mouth. There was no postoperative difficulty in the follow-up phase of 14 months. In contrast to the present case that was followed during four years, those cases were followed in the course of less than one and a half years; however, one of the difficulties of 3D custom-made porous titanium plates is that the long-term tolerance is not recognized, but it can be assumed that the tolerance will undoubtedly not be inferior to that of traditional plates [3, 11]. Even though the alloplastic graft utilized in this case was 3D CAD/CAM porous titanium, these mechanics do not remove the probability of exposure; therefore, it is essential to assess patient's tissues and, most importantly, evade the symphysis area [3, 11, 12].

The present case, together with the cases previously described by Qassemyar et al. [3] and Touré and Gouet [5], has used dental implants. The different advantages presented by this kind of 3D custom-made porous titanium plates for complete reconstructions together with dental implants are relevant [3, 5, 13, 14]. A meticulous oral-dental follow-up is crucial for the stability of these procedures [5, 13, 15].

The 3D custom-made porous titanium plates for bone reconstructions are simple, rapid, and predictable. Moreover, dental rehabilitation does not demand an osseointegration stage.

## Data Availability

The clinical data utilized in this report are described in this article.
